# Supplementary material for: Design, Synthesis, Antibacterial, Antifungal and Anticancer Evaluations of Novel β-Pinene Quaternary Ammonium Salts
Source: Int J Mol Sci. 2021 Oct 19;22(20):11299. doi: 10.3390/ijms222011299 (PMC8539267; doi:10.3390/ijms222011299)
Supplement: Supplementary file 1 [file ijms-22-11299-s001.zip › ijms-1416844-supplementary.pdf]

Supplementary Materials

Design, Synthesis, Antibacterial, Antifungal and Anticancer Evaluations of Novel *β*-Pinene Quaternary Ammonium Salts

Li Zhang [1,†], Xue-Zhen Feng [2,†], Zhuan-Quan Xiao [3], Guo-Rong Fan [1], Shang-Xing Chen [1], Sheng-Liang Liao [1], Hai Luo [1,*] and Zong-De Wang [1,*]

1 East China Woody Fragrance and Flavor Engineering Research Center of National Forestry and Grassland Administration, Camphor Engineering Research Center of National Forestry and Grassland Administration/Jiangxi Province, College of Forestry, Jiangxi Agricultural University, Nanchang 330045, China; zhangli_miaomuli@163.com (L.Z.); fgr1971@163.com (G.-R.F.); csxing@126.com (S.-X.C.); liaosl@jxau.edu.cn (S.-L.L.)
2 National Engineering Laboratory for Biomass Chemical Utilization, Institute of Chemical Industry of Forest Products, Chinese Academy of Forestry, Nanjing 210042, China; xuezhen__feng@163.com
3 College of Chemistry, Jiangxi Normal University, Nanchang 330022, China; xiaozhq3501@163.com
* Correspondence: luohai87@126.com (H.L.); jxzongdewang@163.com (Z.-D.W.)
† The first two authors contributed equally to the work.

Table S1. The antifungal activities of *β*-Pinene quaternary ammonium salts against ten fungi.

Com.	Fungi	Testing the concentration (μg/mL)							EC_{50}	R^2	Toxic regression equation
		200.00	100.00	50.00	25.00	12.50	6.25	3.13			
4a	A	99.50	96.28	95.04	80.40	74.44	60.09	43.20	4.50	0.9870	y=4.0474+1.4589x
	B	100.00	100.00	98.24	78.19	53.08	33.80		10.92	0.9585	y=0.7439+4.0966x
	C	96.01	81.05	69.83	54.61	23.19			27.09	0.9860	y=2.2715+1.9043x
	D	99.77	94.48	91.49	73.56	58.85	43.24		9.45	0.9760	y=3.1591+1.8869x
	E	85.57	70.63	59.49	41.27	33.67			31.98	0.9910	y=3.1360+1.2386x
	F	99.21	90.81	83.20	69.82	44.88			15.09	0.9810	y=2.6912+1.9587x
	G	100.00	100.00	100.00	75.40	35.29	20.77		10.82	0.9214	y=0.1384+4.6998x
	H	83.33	82.54	85.71	84.39	14.55			18.01	0.9830	y=3.3433+1.3418x
	I	98.85	92.20	86.01	75.46	68.12	59.33	29.78	6.34	0.9780	y=3.9164+1.3514x
	J	92.78	77.58	59.54	40.46	23.97			33.76	0.9960	y=2.2919+1.7718x
4b	A	91.47	85.52	71.59	65.18	37.60			17.42	0.9840	y=3.3339+1.3425x
	B	100.00	100.00	94.67	83.11	43.56			15.10	0.9525	y=0.6258+4.7722x
	C	95.38	93.75	72.73	58.81	39.77			18.21	0.9814	y=2.8246+1.7260x
	D	100.00	100.00	93.13	79.62	52.13	39.57		10.97	0.9354	y=0.8710+3.9692x
	E	86.95	76.24	50.28	45.30	40.88			27.83	0.9490	y=3.3005+1.1764x
	F	99.54	95.20	77.60	63.20	52.27	49.07		10.78	0.9464	y=3.1980+1.7448x
	G	100.00	99.09	82.27	72.27	40.23			18.85	0.9422	y=0.1963+4.0745x
	H	100.00	100.00	100.00	68.30	25.13			13.71	0.8820	y=0.9929+5.2702x
	I	100.00	100.00	96.08	93.21	83.29	59.53	43.28	5.39	0.9361	y=2.7575+3.0642x
	J	92.50	86.70	70.64	59.17	38.53			18.98	0.9961	y=3.1561+1.4426x
4c	A	83.19	76.34	59.44	39.44	17.18			40.82	0.9873	y=2.4300+1.5954x
	B	100.00	100.00	100.00	76.53	39.91	21.36		10.54	0.9224	y=0.2423+4.6520x
	C	99.29	93.75	72.73	58.81	39.77			19.58	0.9822	y=2.1101+2.2371x
	D	100.00	100.00	95.71	85.10	64.90	47.73		9.36	0.9396	y=1.329+3.7788x

	E	65.94	56.25	42.61	32.39	14.77		77.68	0.9861	y=2.7843+1.1721x
	F	83.45	70.03	60.47	53.49	44.19		20.67	0.9834	y=3.8316+0.8883x
	G	99.81	98.67	91.33	65.56	49.78		14.32	0.9938	y=2.0750+2.5301x
	H	100.00	100.00	100.00	91.23	32.21		11.02	0.8944	y=0.0437+4.8396
	I	97.49	91.71	82.90	78.24	52.85	36.27	10.25	0.9915	y=3.4976+1.4864x
	J	98.76	93.75	72.92	58.75	32.71		21.13	0.9931	y=2.0523+2.2250x
4d	A	85.60	63.32	43.21	31.52	23.37		49.63	0.9750	y=2.5216+1.4615x
	B	52.25	28.50	12.00	10.50	4.25		229.37	0.9740	y=1.6727+1.4095x
	C	85.28	60.15	46.70	28.93	19.54		52.94	0.9840	y=2.3513+1.5386x
	D	96.50	75.27	66.96	51.20	35.01		24.62	0.9600	y=2.6676+1.6764x
	E	14.95	5.93	3.61	0.77	1.03		113.18	0.9910	y=2.1440+1.3906x
	F	89.08	64.56	45.87	33.50	21.36		46.15	0.9870	y=2.3193+1.6108x
	G	28.54	13.93	7.08	2.05	4.11		906.84	0.9070	y=1.7565+1.0967x
	H	58.98	16.99	7.04	/	/		181.88	0.9755	y=1.3802+2.8234x
	I	93.13	67.94	55.73	42.24	31.55		32.56	0.9550	y=2.6915+1.5259x
	J	92.40	73.53	60.78	39.71	29.66		32.12	0.9850	y=2.5855+1.6024x
4e	A	83.77	63.87	45.55	32.20	21.20		50.53	0.9904	y=2.5178+1.14571x
	B	83.57	49.77	16.90	8.45	4.23		94.06	0.9743	y=0.5602+2.2499x
	C	79.14	67.91	55.88	44.12	34.76		33.31	0.9980	y=3.4729+1.0030x
	D	92.16	87.84	80.59	70.59	45.10		11.77	0.9783	y=3.6825+1.2303x
	E	45.45	28.48	22.73	14.55	8.48		296.61	0.9926	y=2.5316+0.9985x
	F	88.18	77.83	63.05	46.31	15.27		36.81	0.9820	y=2.2550+1.7530x
	G	82.38	54.76	27.62	12.86	6.67		81.94	0.9894	y=1.1135+2.0311x
	H	58.33	32.22	26.11	8.33	6.11		165.09	0.9791	y=1.7338+1.4728x
	I	93.25	73.25	51.75	30.75	21.75		40.37	0.9833	y=1.9727+1.8849x
	J	98.16	73.16	47.63	36.32	23.16		36.03	0.9428	y=1.5816+2.1960x
4f	A	44.92	36.80	30.20	26.90	21.32		391.04	0.9935	y=3.6101+0.5362x
	B	49.56	45.13	34.96	26.99	23.01		189.69	0.9913	y=3.5276+0.6464x
	C	53.03	41.67	31.82	19.70	15.66		168.86	0.9945	y=2.9198+0.9338x

	**D**	**70.29**	**50.59**	**44.41**	**38.24**	**11.18**			**74.83**	**0.9450**	**y=2.6259+1.2668x**
	**E**	**17.20**	**12.37**	**10.48**	**8.06**	**6.45**			**26411.41**	**0.9926**	**y=2.9613+0.4610x**
	**F**	**75.12**	**65.61**	**51.22**	**25.37**	**11.71**			**61.94**	**0.9867**	**y=2.1423+1.5947x**
	**G**	**47.02**	**35.78**	**22.94**	**13.76**	**5.05**			**203.90**	**0.9933**	**y=2.0407+1.2814x**
	**H**	**41.11**	**35.93**	**28.15**	**20.74**	**11.11**			**306.31**	**0.9808**	**y=2.9790+0.8129x**
	**I**	**59.15**	**49.79**	**45.96**	**31.91**	**20.43**			**93.84**	**0.9801**	**y=3.3092+0.8572x**
	**J**	**41.67**	**20.83**	**14.72**	**10.00**	**5.00**			**398.38**	**0.9617**	**y=2.1164+1.1089x**
	**A**	**65.38**	**64.36**	**58.97**	**58.68**	**57.96**	**55.32**	**44.28**	**3.98**	**0.9154**	**y=4.8496+0.2507x**
	**B**	**100.00**	**100.00**	**100.00**	**100.00**	**100.00**	**55.81**	**32.47**	**1.81**	**0.7978**	**y=4.2020+3.0936x**
	**C**	**69.13**	**63.56**	**55.49**	**47.12**	**43.99**			**27.09**	**0.9915**	**y=4.1811+0.5715x**
	**D**	**100.00**	**100.00**	**100.00**	**100.00**	**100.00**	**93.08**	**86.00**	**0.43**	**0.7974**	**y=5.8195+2.2302x**
**Chlorothalonil**	**E**	**100.00**	**100.00**	**100.00**	**100.00**	**100.00**	**33.36**	**21.99**	**2.41**	**0.7948**	**y=3.7253+3.3435x**
	**F**	**82.40**	**73.95**	**73.19**	**72.54**	**66.63**	**63.14**	**59.18**	**0.67**	**0.9657**	**y=5.805+0.3438x**
	**G**	**100.00**	**100.00**	**100.00**	**100.00**	**100.00**	**45.98**	**42.98**	**1.74**	**0.7916**	**y=4.2630+3.0534x**
	**H**	**100.00**	**100.00**	**100.00**	**100.00**	**100.00**	**100.00**	**59.39**	**0.07**	**0.6121**	**y=3.8390+5.1823x**
	**I**	**91.14**	**90.13**	**89.37**	**89.11**	**78.73**	**77.68**	**74.39**	**0.09**	**0.9385**	**y=5.4523+0.4256x**
	**J**	**100.00**	**100.00**	**100.00**	**100.00**	**100.00**	**50.96**	**25.03**	**2.05**	**0.7984**	**y=4.0043+3.2005x**

Note: A: *Fusarium oxysporum f.sp. niveum* (*F. oxysporum f.sp. niveum*), B: *Phytophthora nicotianae var.nicotianae* (*P. nicotianae var.nicotianae*), C: *Colletotrichum acutatum* (*C. acutatum*), D: *Rhizoctonia solani* (*R. solani*) , E: *Coriolus versicolor* (*C. versicolor*) , F: *Fusarium verticillioides* (*F. verticillioides*), G: *Diplodia pinea* (*D. pinea*), H: *Poria vaporaria*(*P. vaporaria*), I: *Fusicoccumaesculi*, J: *Colletotrichum gloeosprioides* (*C. gloeosprioides)*

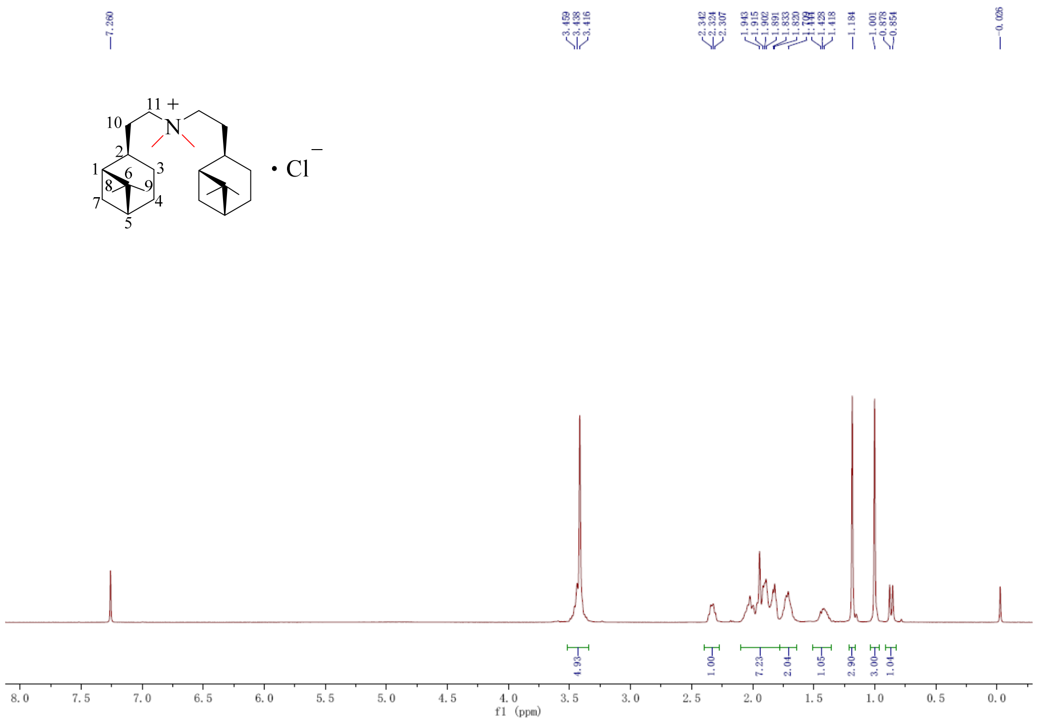

Figure S1. The ^{1}H NMR spectra of Compound 4a.

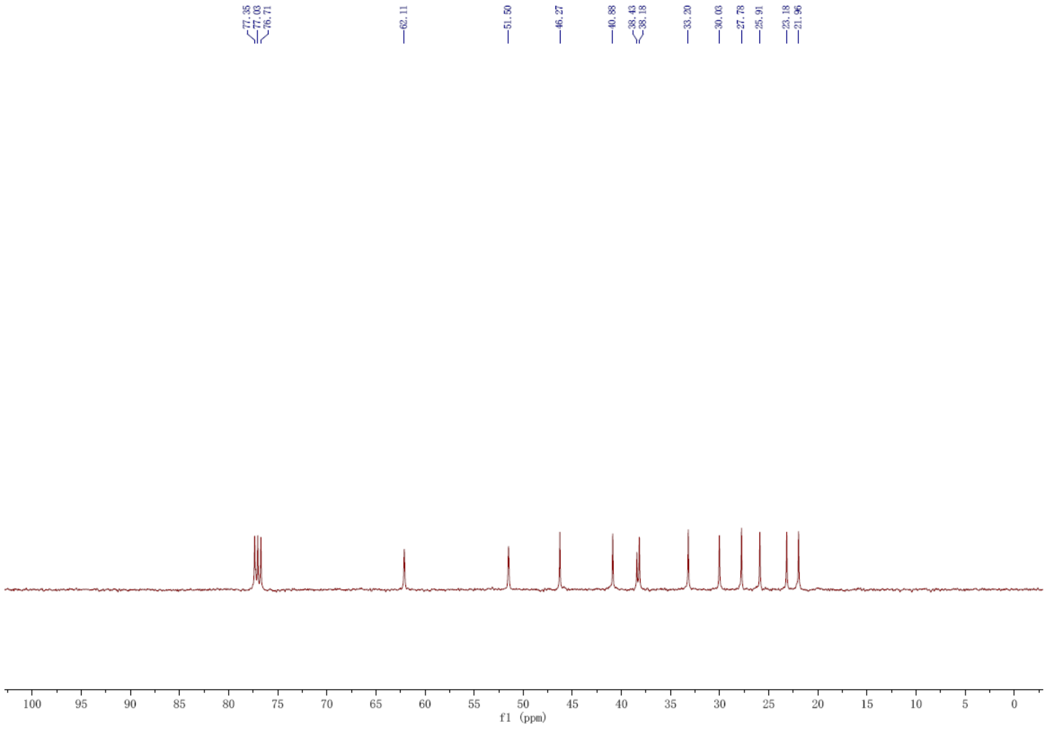

Figure S2. The ^{13}C NMR spectra of Compound 4a.

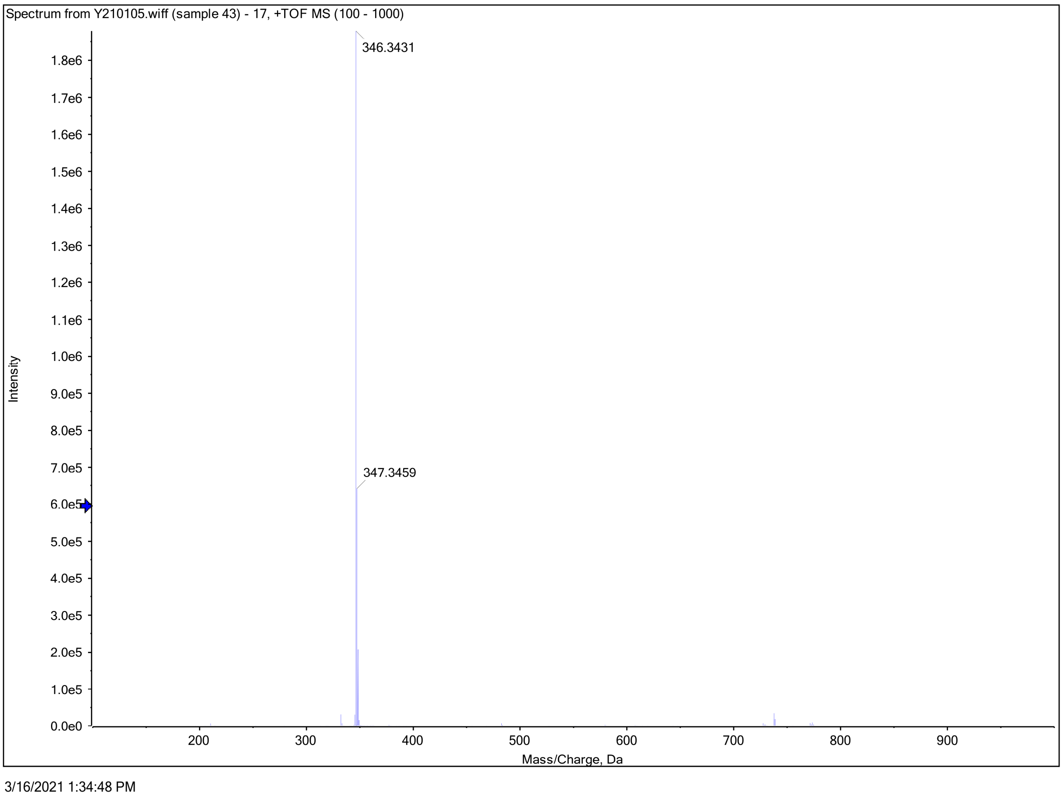

Figure S3. The HRMS spectra of Compound 4a.

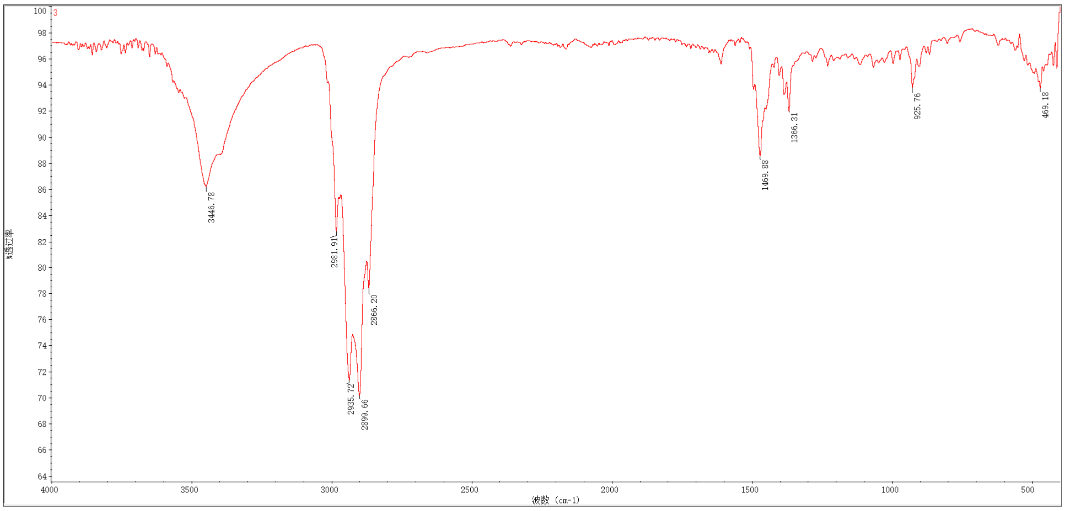

Figure S4. The FT-IR spectra of Compound 4a.

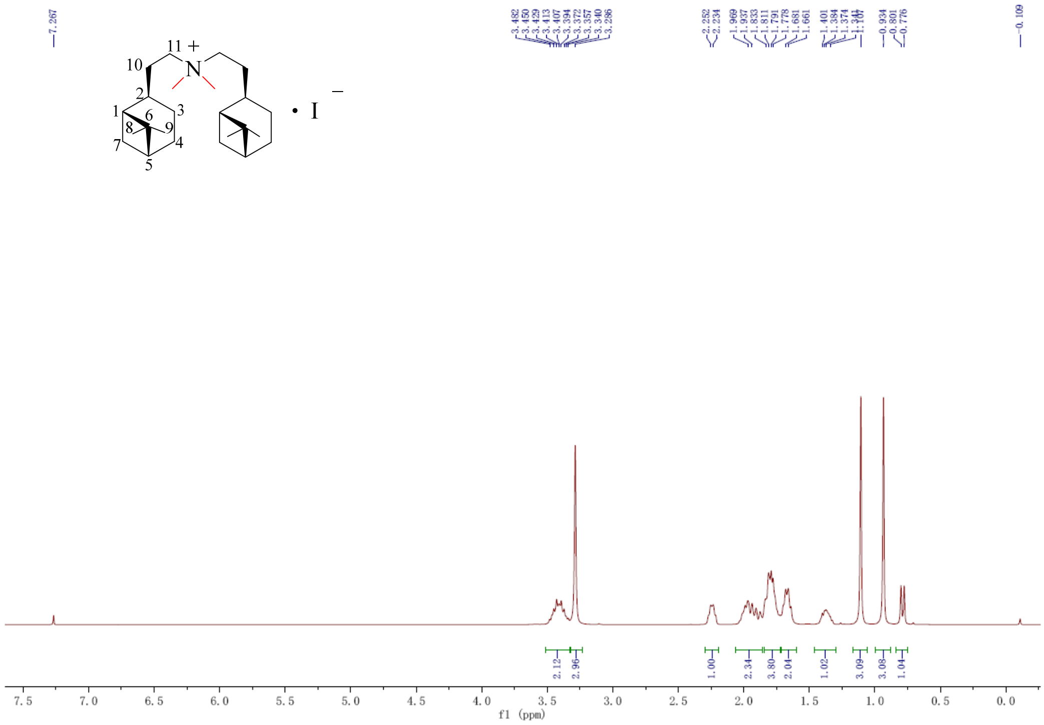

Figure S5. The ^{1}H NMR spectra of Compound 4a.

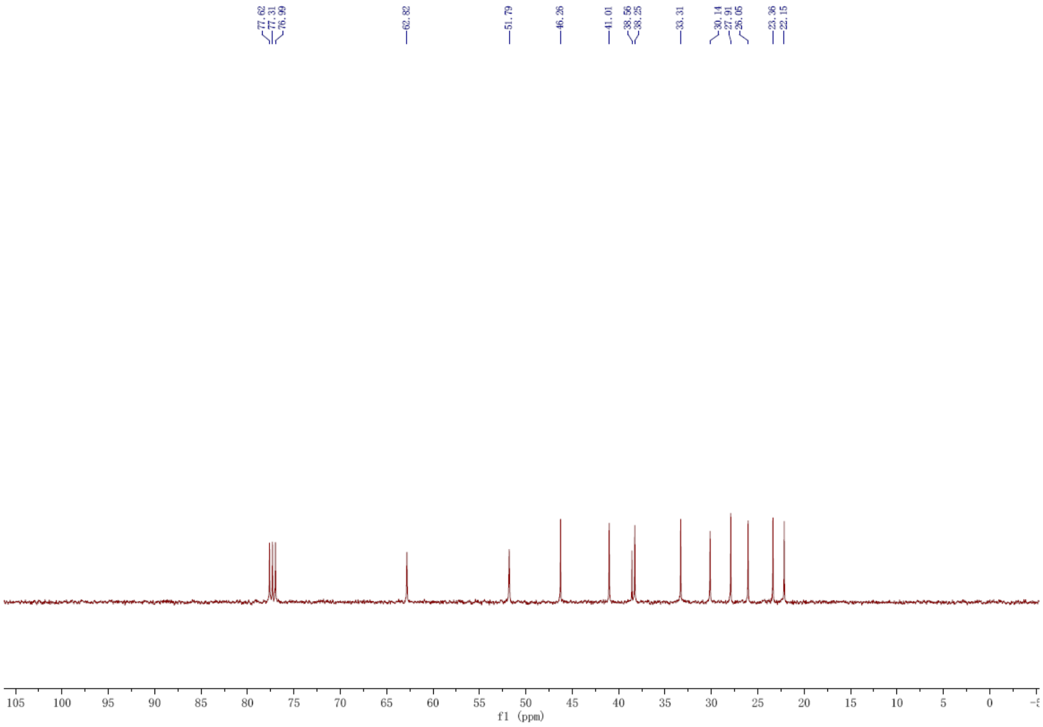

Figure S6. The ^{13}C NMR spectra of Compound 4b.

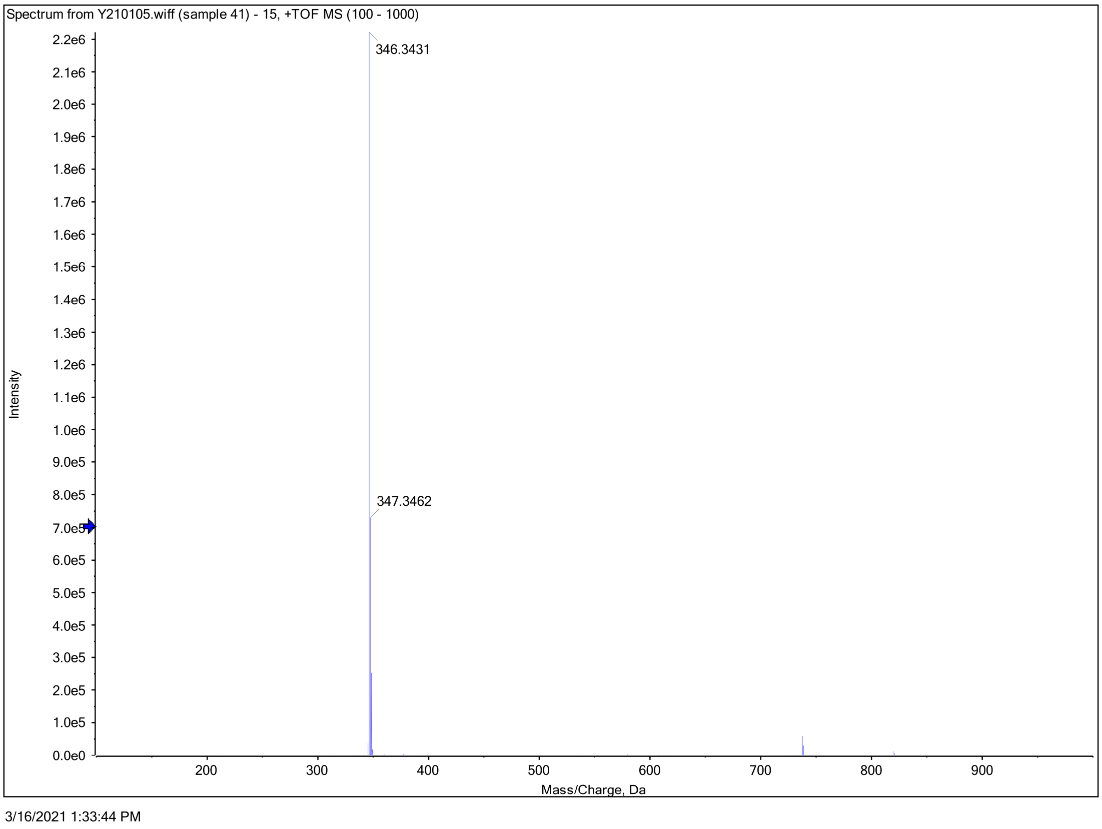

Figure S7. The HRMS spectra of Compound 4b.

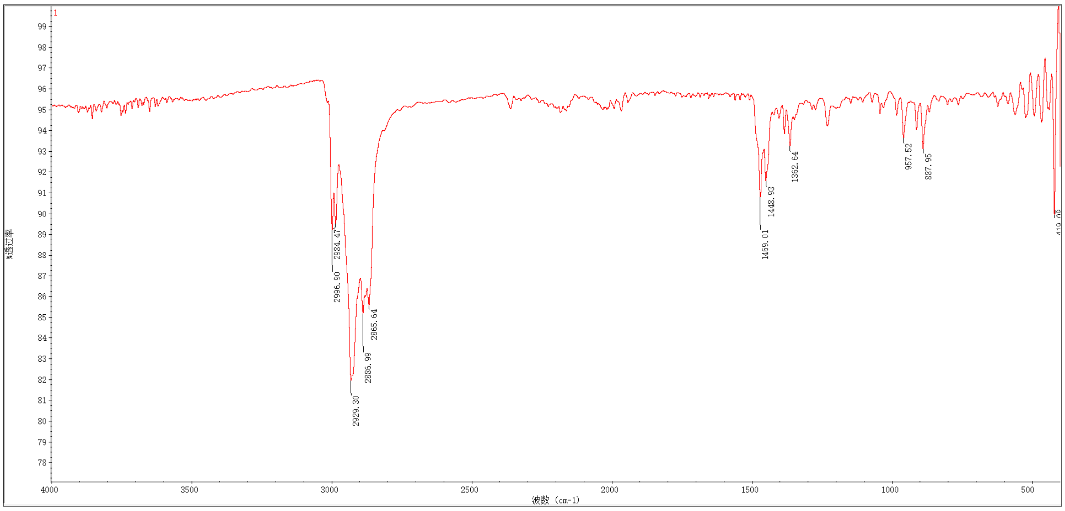

Figure S8. The FT-IR spectra of Compound 4b.

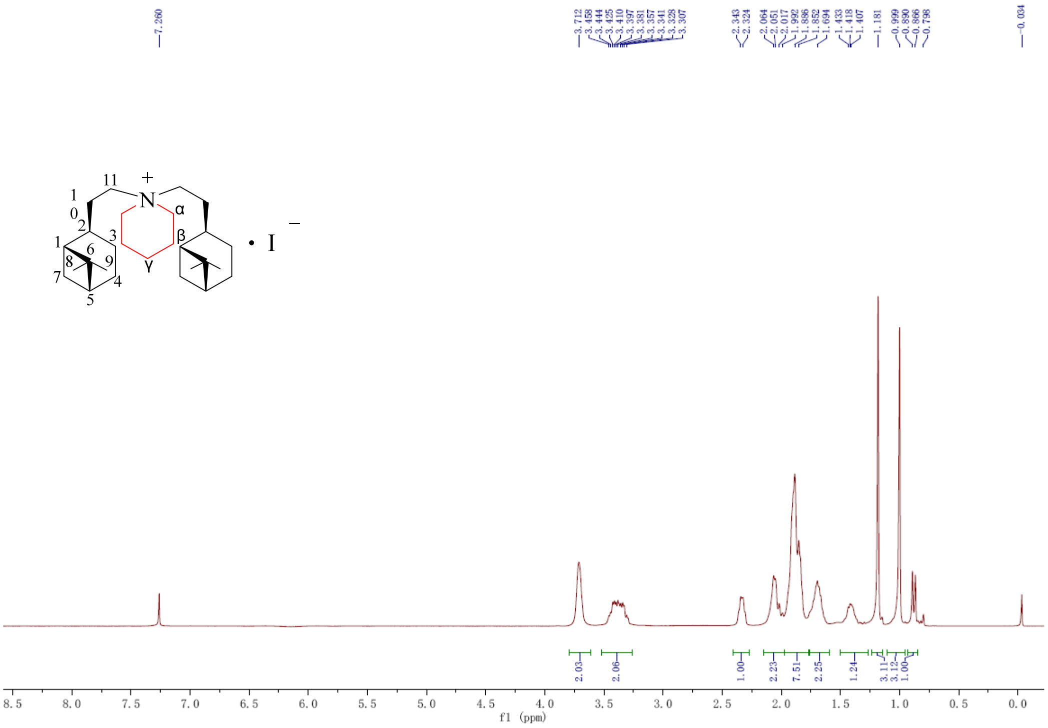

Figure S9. The ^{1}H NMR spectra of Compound 4c.

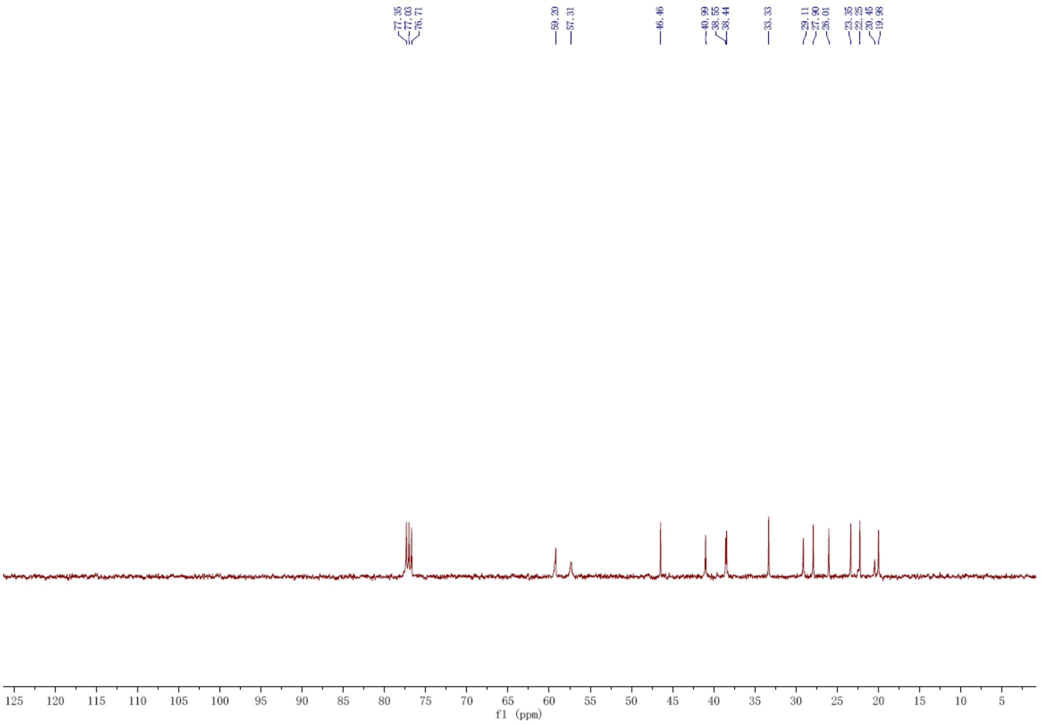

Figure S10. The ^{13}C NMR spectra of Compound 4c.

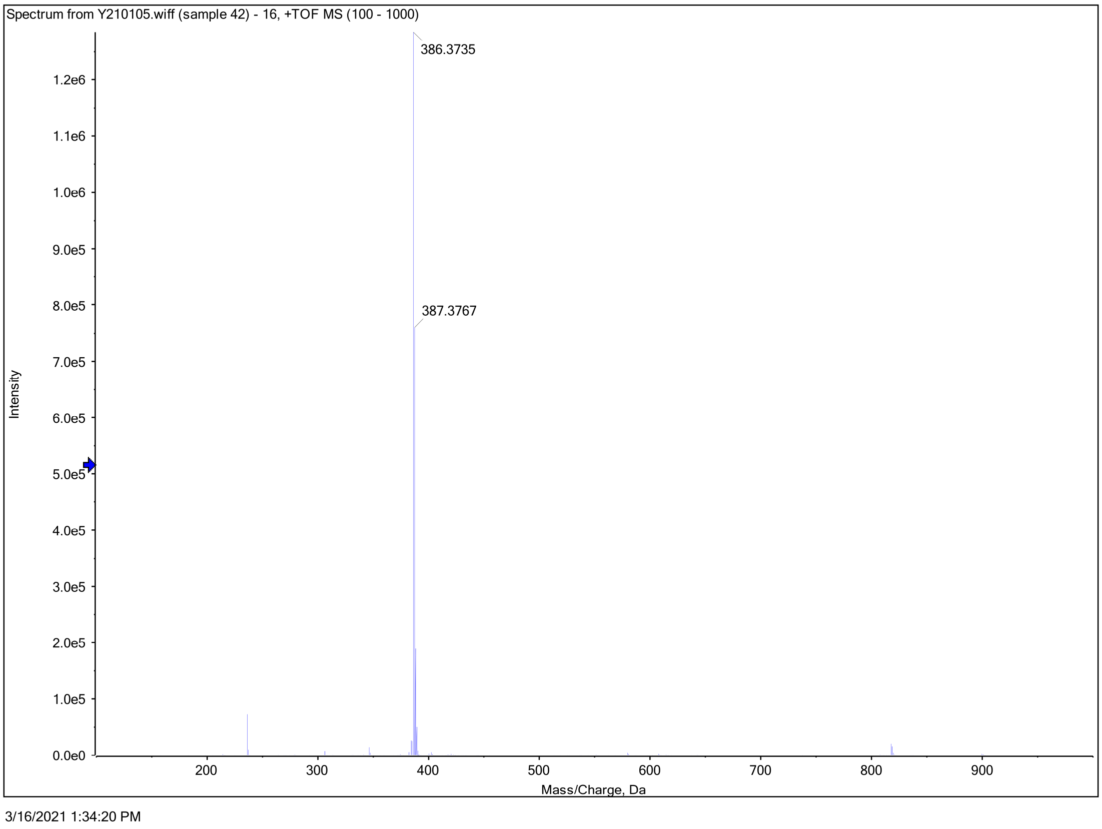

Figure S11. The HRMS spectra of Compound 4c.

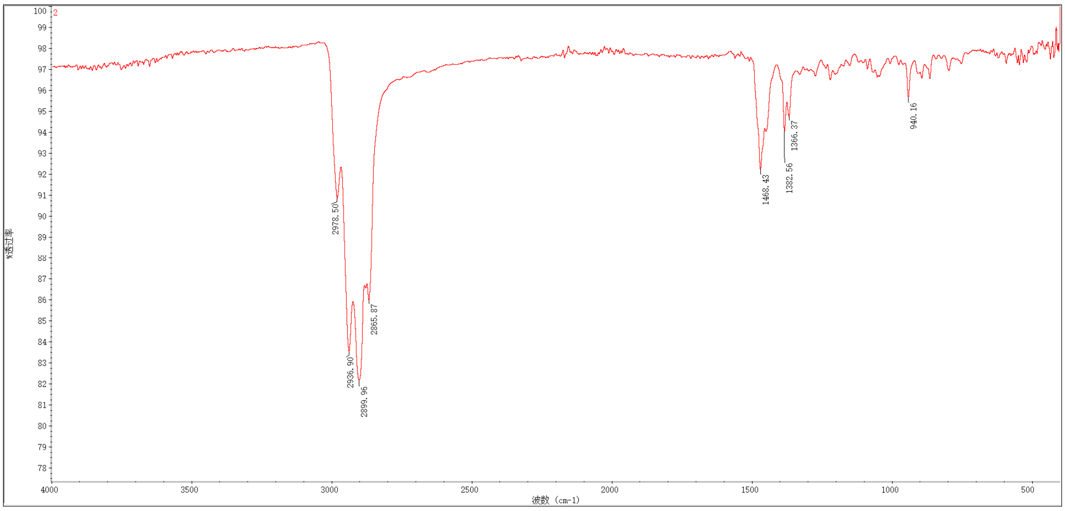

Figure S12. The FT-IR spectra of Compound 4c.

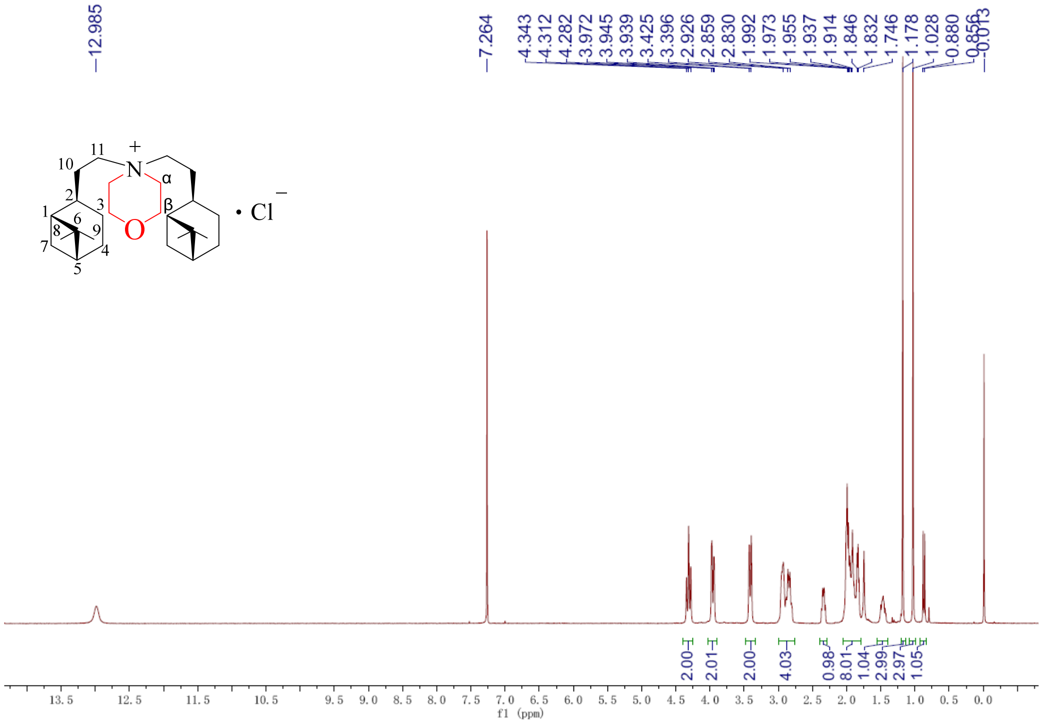

Figure S13. The ^{1}H NMR spectra Compound 4d.

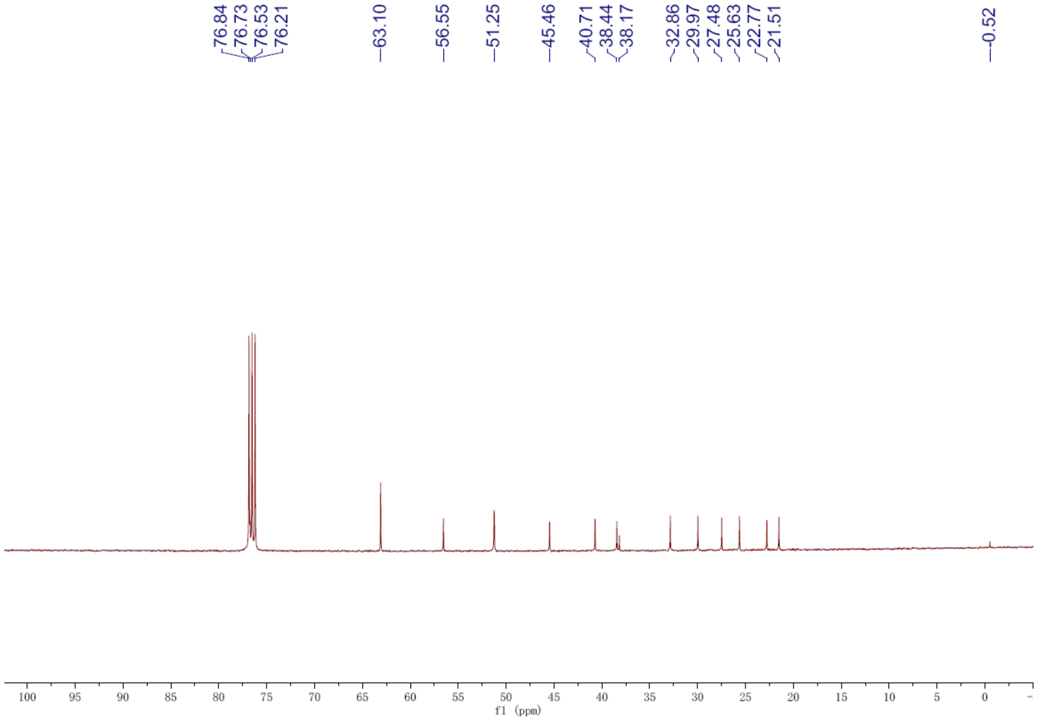

Figure S14. The ^{13}C NMR spectra of Compound 4d.

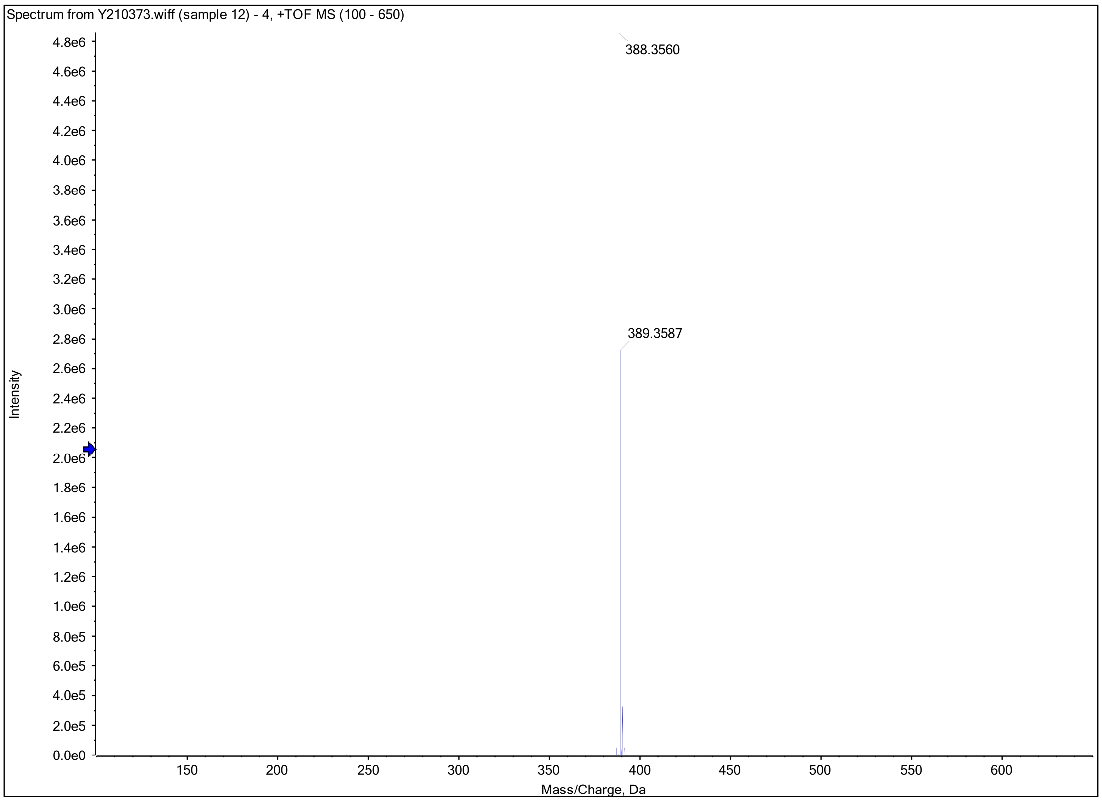

6/30/2021 4:27:34 PM

Figure S15. The HRMS spectra of Compound 4d.

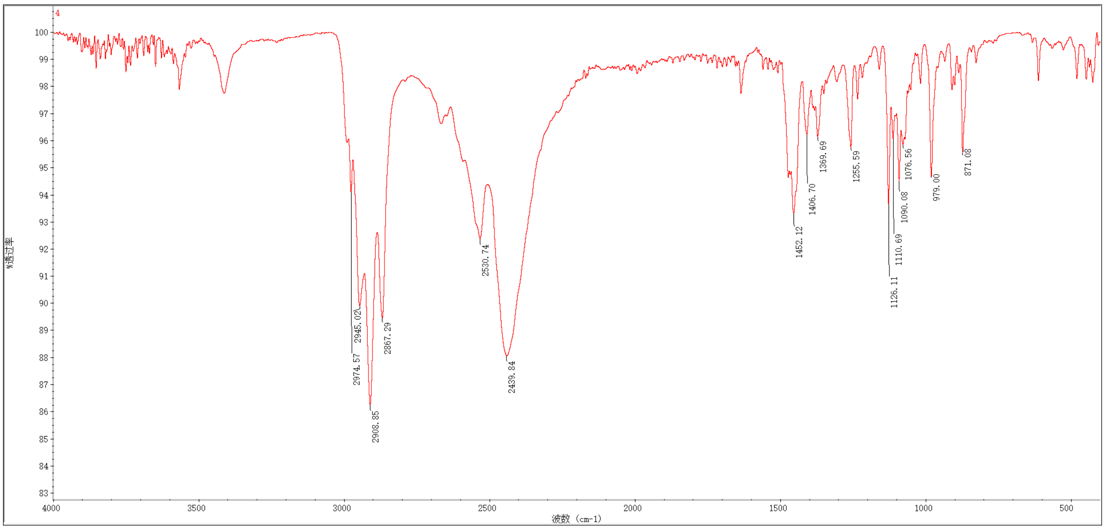

Figure 16. The FT-IR spectra of Compound 4d.

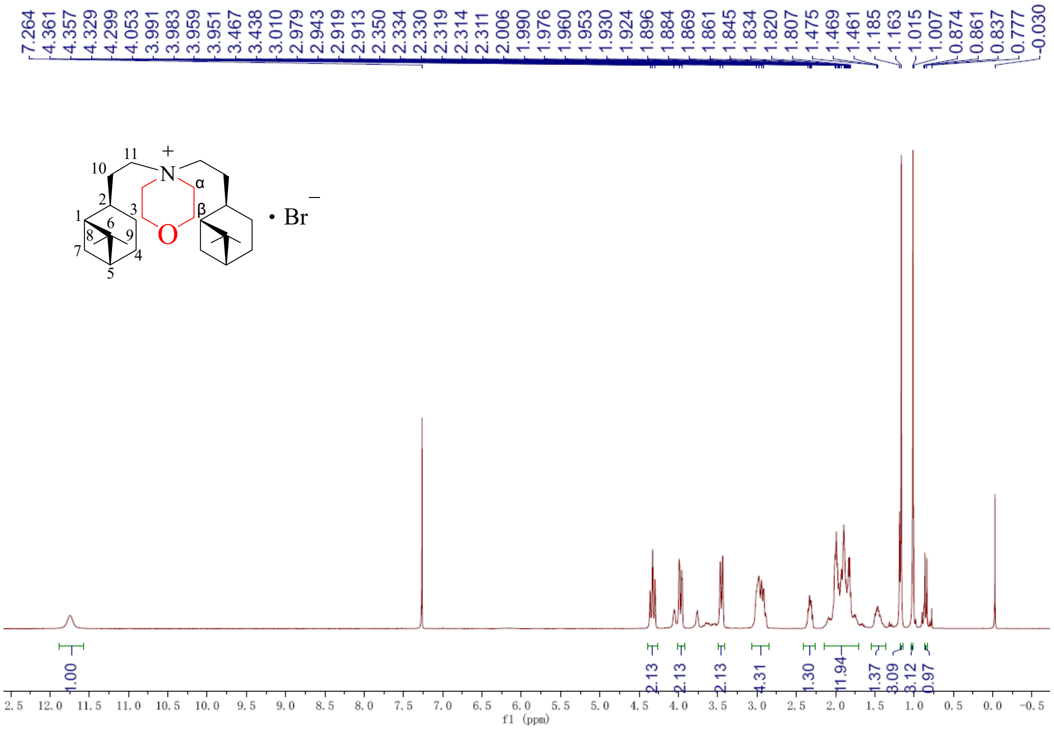

Figure S17. The ^{1}H NMR spectra Compound 4e.

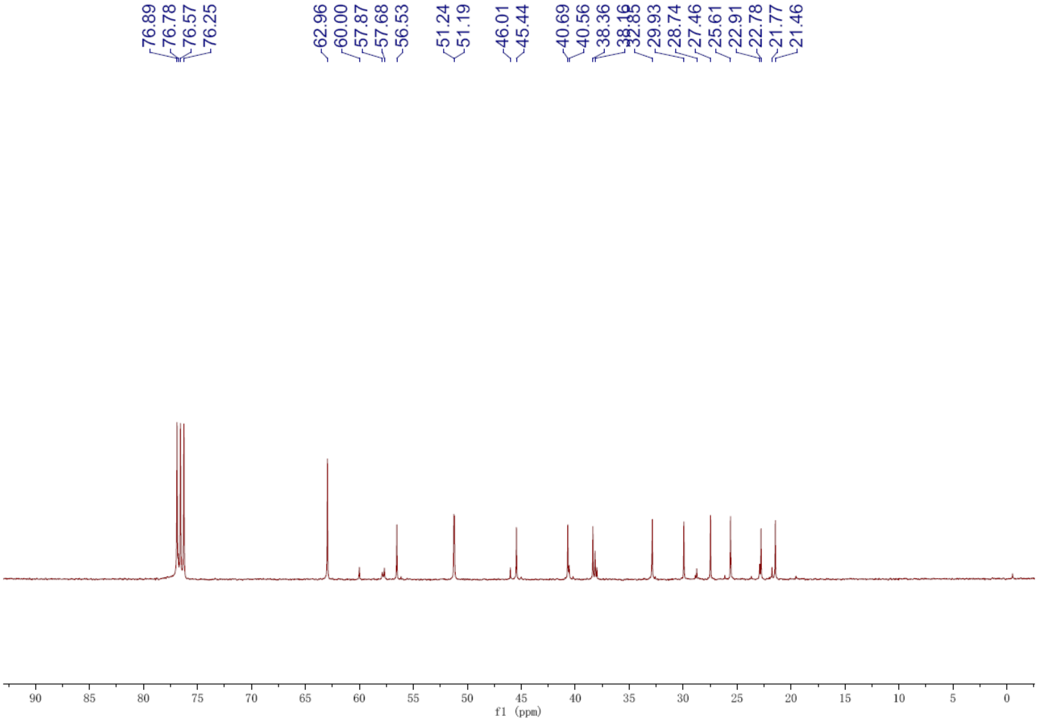

Figure S16. The ^{13}C NMR spectra of Compound 4e.

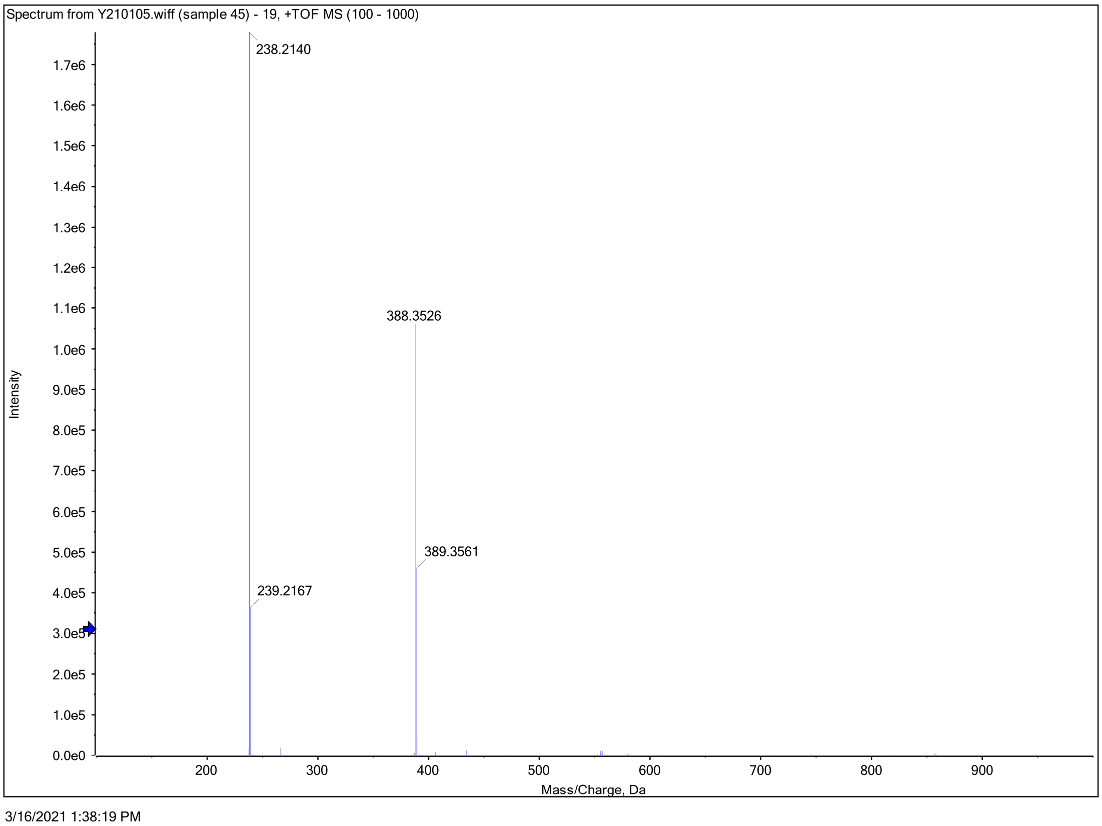

Figure S19. The HRMS spectra of Compound 4e.

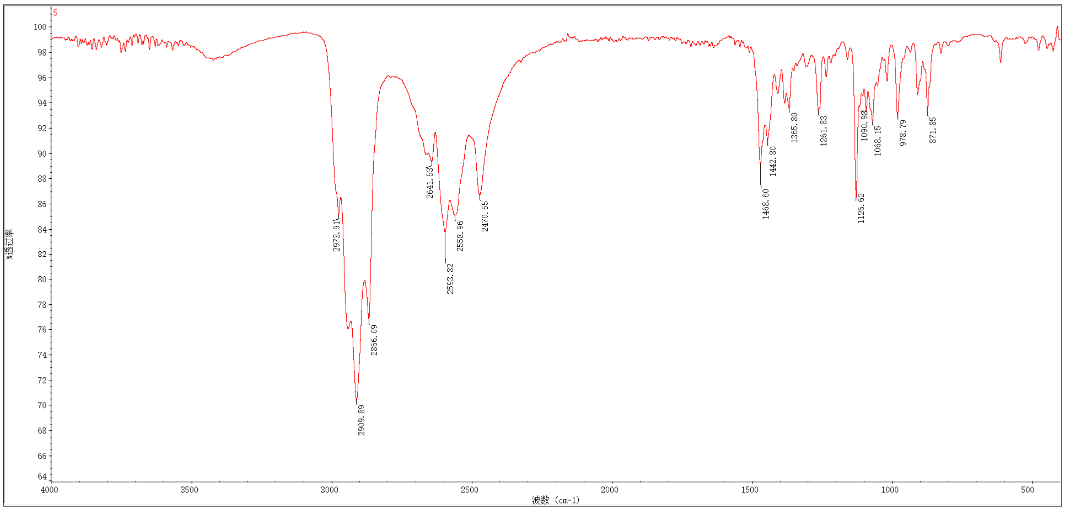

Figure S20. The FT-IR spectra of Compound 4e.

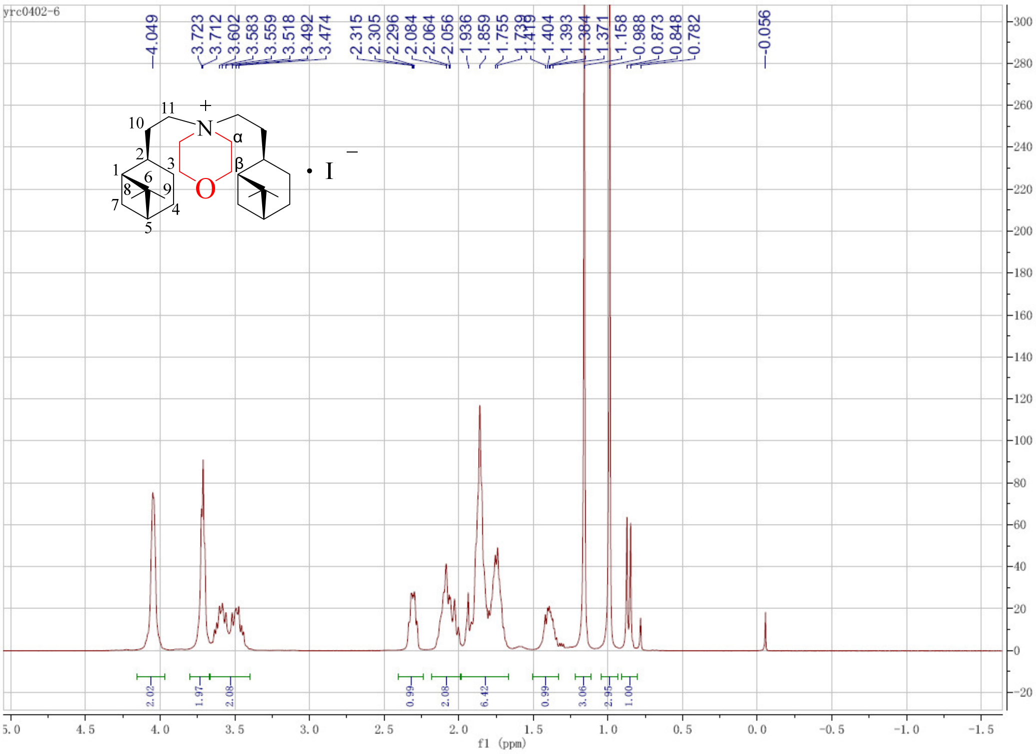

Figure S21. The ^{1}H NMR spectra Compound 4f.

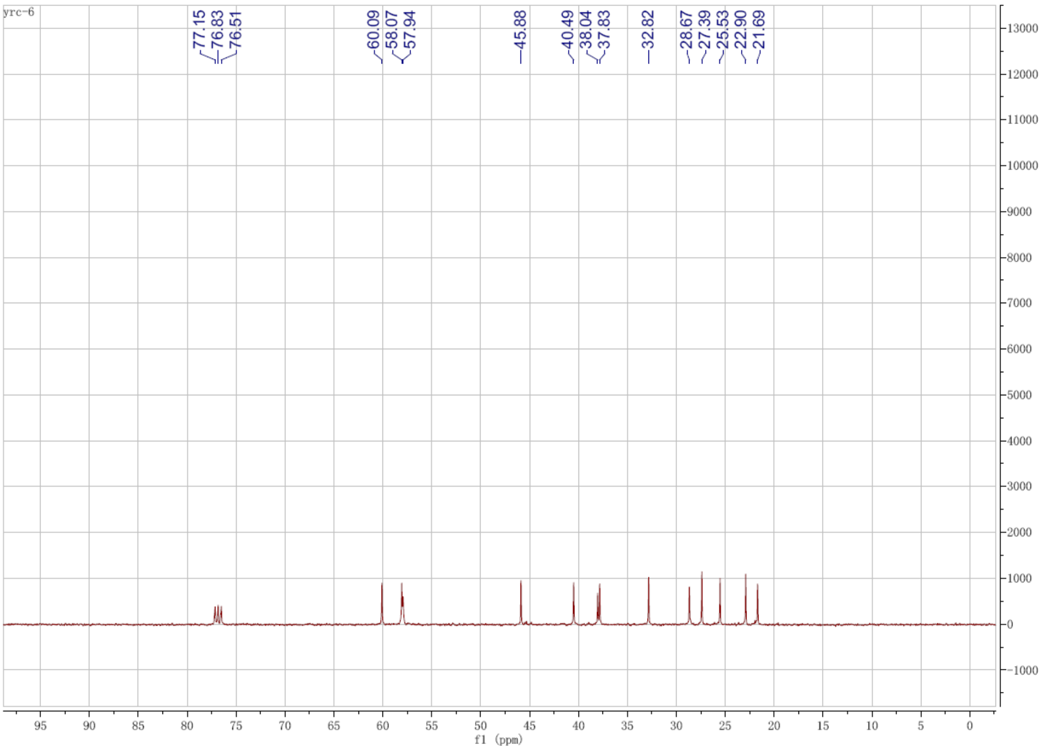

Figure S22. The ^{13}C NMR spectra of Compound 4f.

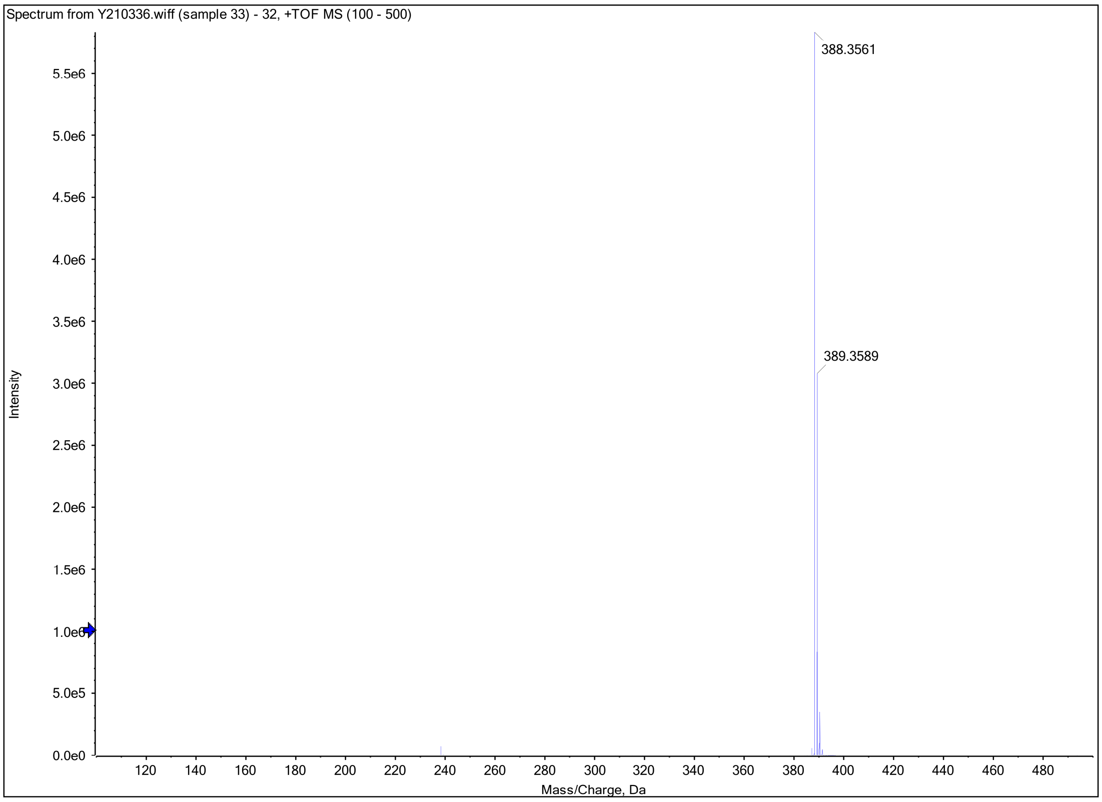

6/11/2021 6:38:34 PM

Figure S23. The HRMS spectra of Compound 4f.

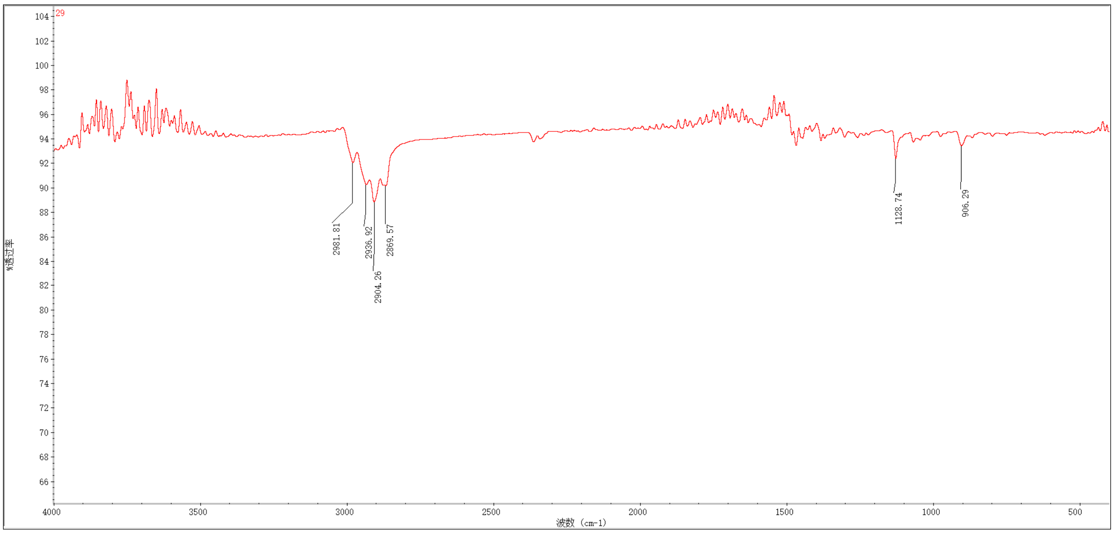

Figure S24. The FT-IR spectra of Compound 4f.
